# Mechanism of Disease: Recessive ADAMTSL4 Mutations and Craniosynostosis with Ectopia Lentis

**DOI:** 10.1155/2022/3239260

**Published:** 2022-03-26

**Authors:** Jonas Gustafson, Maria Bjork, Conny M. A. van Ravenswaaij-Arts, Michael L. Cunningham

**Affiliations:** ^1^Seattle Children's Research Institute, Center for Developmental Biology and Regenerative Medicine, Seattle, WA, USA; ^2^Närhälsan Ågårdsskogens Vårdcentral, Lidköping, Sweden; ^3^University of Groningen, University Medical Center Groningen, Department of Genetics, Groningen, Netherlands; ^4^Seattle Children's Hospital Craniofacial Center, Seattle, WA, USA; ^5^University of Washington, Department of Pediatrics, Seattle, WA, USA

## Abstract

Craniosynostosis, the premature fusion of the calvarial bones, has numerous etiologies. Among them, several involve mutations in genes related to the TGFb signaling pathway, a critical molecular mediator of human development. These TGFb pathway-associated craniosynostosis syndromes include Loeys–Dietz syndrome (LDS) and Shprintzen–Goldberg syndrome (SGS). LDS and SGS have many similarities common to fibrillinopathies, specifically Marfan syndrome (MFS), which is caused by mutations in FBN1. Historically discriminating features of MFS from LDS and SGS are (1) the presence of ectopia lentis (the subluxation/dislocation of the ocular lens) and (2) the absence of craniosynostosis. Curiously, several instances of a seemingly novel syndrome involving only craniosynostosis and ectopia lentis have recently been reported to be caused by recessive mutations in ADAMTSL4, a poorly characterized gene as of yet. Here, we report on two new cases of craniosynostosis with ectopia lentis, each harboring recessive mutations in ADAMTSL4. We also discuss a proposed mechanism for the relationship between ADAMTSL4, FBN1, and TGFb pathway-related syndromes.

## 1. Introduction

### 1.1. Craniosynostosis

At birth, the calvaria of the human skull are normally separated by soft, membranous sutures to allow for deformation of the skull during birth and subsequent brain growth. Craniosynostosis (CS) is defined by the premature ossification and subsequent fusion of these sutures. CS occurs in between 4.3 [1] and 7.2 [2] per 10,000 live births worldwide. While CS occasionally manifests as part of a characterized syndrome (Apert Syndrome, Crouzon Syndrome, Saethre–Chotzen Syndrome, etc.) in approximately 15% of cases [1, 3], isolated CS is a far more common diagnosis. Among isolated cases, only ∼15–18% have a known (or suspected) genetic etiology [4, 5]. Research suggests that environmental factors can also influence susceptibility to CS. These factors include multiple gestation pregnancies and other sources of intrauterine constraint (primiparity and high birth weight) [6] as well as causes of attenuated sutural strain such as shunted hydrocephalus [7].

### 1.2. TGFb Pathway-Associated Syndromes and CS

In brief, the canonical TGFb pathway is a complex signaling cascade that regulates a myriad of cellular functions including proliferation, differentiation, migration, and apoptosis. It is composed of secreted ligands (TGFb 1–3) which bind to a heteromeric receptor complex (TGFBR1 and TGFBR2) and subsequently trigger SMAD phosphorylation. Activated receptor SMADs (2 and 3) complex with SMAD4 and translocate to the nucleus where they help regulate gene transcription (Reviewed in [8, 9]). Outside of the more commonly recognized CS syndromes mentioned above, CS is also a recurring phenotype in many TGFb pathway-associated connective tissue disorders, specifically Loeys–Dietz Syndrome 1 and 2 (OMIM #609192 and OMIM #610168, respectively) and Shprintzen–Goldberg Syndrome (SGS) (OMIM #182212). These syndromes share an overlapping array of phenotypes including cardiovascular anomalies, joint abnormalities, and dermal dysmorphology and are often distinguished in diagnosis by the gene harboring the causal mutation (TGFBR1 and TGFBR2 in LDS 1 and 2, respectively, and SKI in SGS). While SGS and LDS tend to phenocopy common fibrillinopathies, specifically Marfan syndrome, there are historically discriminating phenotypes that help inform diagnoses. One of these discriminating phenotypes is craniosynostosis which is common in some types of LDS (LDS1 and LDS2) and is a hallmark symptom of SGS (originally referred to as “Marfanoid craniosynostosis”) but is not considered a phenotype associated with classic MFS [10]. Additionally, MFS often involves ectopia lentis which is traditionally not seen in LDS or SGS [10].

### 1.3. Ectopia Lentis

Ectopia lentis is defined as the detachment of the ocular lens from the ciliary body as a result of the disintegration or disruption of the zonule fibers that hold it in place (Reviewed in [11]). This can occur through physical trauma to the eye or through genetic mutations which can cause isolated ectopia lentis (IEL), ectopia lentis et pupillae (ELP), or EL as part of a more complex disorder (such as MFS, Weill–Marchesani syndrome (WMS), and homocystinuria). Mutations in FBN1 [12], CBS [13], ADAMTS10 [14], ADAMTSL4 [15], ADAMTS17 [16], LTBP2 [17], COL18A1 [18], PAX6 [19], VSX2 [20], and LEPREL1 [21] have all been described as causal of EL. The physical manifestation of genetic EL has been extensively studied in humans and mice, and it has been shown that the main contribution to the failure of zonule formation is fibrillin microfibril disintegration [22–24].

### 1.4. Craniosynostosis and Ectopia Lentis

Although CS and EL are common features of otherwise phenotypically overlapping syndromes (MFS, LDS, SGS, and WMS), very few cases have been reported in the literature of craniosynostosis and ectopia lentis occurring together. Of those which have been reported, fewer still have been solved with genetic testing. However, all of the cases which have been genetically solved fall into two distinct groups; CS/EL that is part of a particularly severe (neonatal/early onset) case of Marfan syndrome and caused by a dominant mutation in FBN1 [25, 26] or CS/EL that is otherwise isolated and is attributed to recessive mutations in a gene that is comparatively not well understood, ADAMTSL4 [27–29].

### 1.5. ADAMTSL4

ADAMTSL4 (A disintegrin and metalloproteinase with thrombospondin motifs-like 4) is one of 26 proteins that make up the greater ADAMTS(L) family and one of seven of which lack the metalloprotease domain (hence using the “-Like” terminology) (Reviewed in [30]). The complete function of ADAMTSL4 has yet to be elucidated, but it is well established that recessive mutations in ADAMTSL4 cause IEL/ELP and, rarely, EL with CS. ADAMTSL4 is widely expressed in fetal and adult human tissue [15] (GTEx) but has been studied most extensively in the eye due to its association with EL [31]. Gabriel et al. showed that ADAMTSL4 colocalizes with FBN1 and that greater deposition of FBN1 microfibrils was seen in fetal bovine nuchal ligament fibroblast cultures when exposed to exogenous ADAMTSL4 [31]. Proteins related to ADAMTSL4 (ADAMTS10 and ADAMTS17) are also associated with EL suggesting that the ADAMTS(L) family is important in proper zonule formation and also that the ADAMTS(L) proteins do not compensate for each other in vivo.

Here, we report on two new individuals with the rare phenotypic combination of ectopia lentis and craniosynostosis who harbor recessive mutations in ADAMTSL4. Further, we discuss the functional interaction of ADAMTSL4 and FBN1 and hypothesize on how disruptions in each of these proteins might cause the same complex phenotype.

## 2. Materials and Methods

### 2.1. Editorial Policies and Ethical Considerations

This study was approved by Seattle Children's Research Institute (IRB 12394). Informed consent from all human subjects was obtained for submission of this manuscript.

### 2.2. Variant Sequencing

Patient 1 and his parents were consented under protocol number SCH-IRB-12394. DNA was isolated from saliva and the proband's DNA was sent for whole genome sequencing. Results were screened for potentially causal variants in the TGFb pathway and other known CS-associated genes [4]. ADAMTSL4 variants of interest were investigated in parents and validated in the proband by Sanger sequencing.

Patient 2 was introduced to our team after having already undergone clinical sequencing for ADAMTSL4. His parents and sister were consented under protocol number SCH-IRB-12394. DNA was isolated from saliva, and the ADAMTSL4 mutation of interest was investigated in the parents and sister and validated in the proband by Sanger sequencing.

## 3. Case Reports

Patient 1 was born at term via normal spontaneous vaginal delivery after an uncomplicated pregnancy and delivery to a G2P2 mother. His head shape was described as abnormal at birth and upon referral to our center at 3½ weeks of age, he had physical features consistent with bilateral coronal craniosynostosis. Physical examination revealed brachy-turricephaly, retrusion of the frontal bones, moderate retrusion of the surpraorbital rims, anteroposterior enlargement of the anterior fontanelle with moderate supraorbital rim retrusion, and mild proptosis. He had no evidence of midfacial retrusion or extracranial malformations at this age. His length and weight were both at the 95th centile, and his head circumference was at the 50th centile. At four months of age, his mother noted a “shimmering” appearance of his eyes and he was diagnosed with iridodonesis associated with bilateral ectopia lentis. He had developed mild snoring but had no other health concerns. Preoperative computed tomography at nine months of age confirmed the diagnosis of bilateral coronal craniosynostosis with evidence of increased intracranial pressure due to the presence of craniolacunae. There were no concerns about his neurocognitive development, and he met all his developmental milestones. He underwent expansion cranioplasty and fronto-orbital expansion at nine months of age and did well until six years of age when he developed papilledema with recurrent increased intracranial pressure and was treated with a repeat bifrontal advancement surgery. He developed midfacial hypoplasia by age 10, and at skeletal maturity, his height was between the 25–50th centile; however, his weight and head circumference were at the 5th centile. He has developed a significant class II malocclusion which will require bimaxillary advancement surgery. Both parents are phenotypically normal. Maternal family history is negative for craniosynostosis or other birth defects. His father was adopted, and no family history was available.

**Figure 1:**
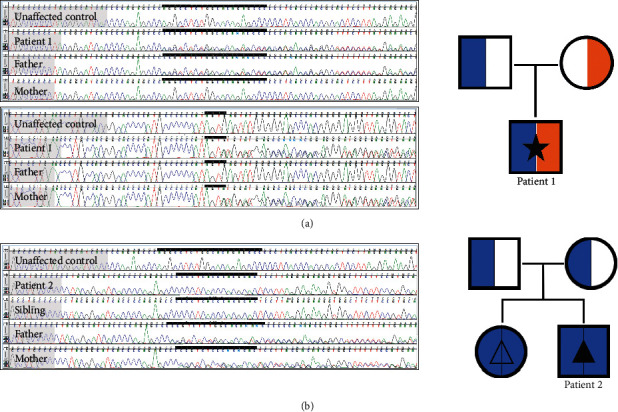
Patient 1. (a) Chromatograms (top: paternally inherited c.767_786del, bottom: maternally inherited c.2177 + 3_2177+6delGAGT). Pedigree (blue: c.767_786del, orange: c.2177 + 3_2177 + 6delGAGT, solid star: ectopia lentis with craniosynostosis). Patient 2. (b) Chromatogram (recessively inherited c.767_786del). Pedigree (blue: c.767_786del, solid triangle: ectopia lentis et pupillae with craniosynostosis, open triangle: isolated ectopia lentis et pupillae).

Two ADAMTSL4 variants, predicted pathogenic by gnomAD, were detected; a 20 base pair deletion (c.767_786del, p.Gln256Profs*∗*38) and a splice site frameshift deletion (c.2177 + 3_2177 + 6delGAGT). The variants were inherited in trans as confirmed by Sanger sequencing (Figure 1(a)).

Patient 2 was born at term via normal spontaneous vaginal delivery after an uncomplicated pregnancy and delivery to a G2P2 mother. He had a scaphocephlaic head shape at birth. His length was above the 97th centile, weight at the 97th centile, and a head circumference above the 97th centile. Ectopia lentis and pupillae were noted at birth. He was diagnosed with sagittal craniosynostosis at five months of age by CT scan and was treated with a cranioplasty at six months of age. At two years of age, he had intraocular lens replacement surgery. He had no other malformations on exam. There were no concerns about his neurocognitive development, and he met all his developmental milestones. Family history was significant for his older sister having ectopia lentis and pupillae. She did not have craniosynostosis, and there is no additional family history of craniosynostosis or other birth defects.

A homozygous 20 bp deletion in ADAMTSL4 (c.767_786del, p.Gln256Profs*∗*38) was identified in the patient and his sister. Sanger sequencing confirmed these results and showed heterozygosity of the variant in each parent (Figure 1(b)).

## 4. Discussion

Craniosynostosis and ectopia lentis are two independent features of several overlapping syndromes, yet curiously very rarely occur together. There are only 2 conditions in which CS and EL seem to co-occur: (1) as symptoms of severe (early onset) FBN1-associated Marfan syndrome and (2) as an otherwise completely isolated phenotypic combination caused by recessive ADAMTSL4 mutations. This suggests an overlap in molecular function between FBN1 and ADAMTSL4 at some level but also differential tissue specificity and regulation.

To date, there have been 10 definitive cases reported in the literature of the co-occurrence of CS and EL. Two of those cases are attributed to dominant mutations in FBN1 and an overall phenotype described as neonatal or early-onset Marfan syndrome (eoMFS) [25, 26]. Four cases are attributed to recessive mutations in ADAMTSL4 and present exclusively with CS and EL [27–29, 32]. (Overwater et al. [29] reports the genotype of one of the monozygotic twins described by Cruysberg et al. [32]. Personal correspondence with the authors confirms that the genotypically unreported twin in fact carries the same genotype). The remaining four cases are genetically “unsolved” [33–36]. Beyond these cases, there are several individuals described in the literature whose phenotype is not as detailed but may be noteworthy. For example, Wojcik et al. [37] describes a patient with confirmed FBN1-associated Marfan syndrome presenting with bilateral lens subluxation, “bulging coronal sutures,” and “findings concerning for craniosynostosis” [37]. Overwater et al. [29] describes a patient with an unknown genetic etiology and a complex phenotype including EL and dolichocephaly, which could represent undiagnosed sagittal CS [29]. Topa et al. [38] reports on a patient with a novel ADAMTSL4 mutation, coronal CS, and vision loss [38]. In this report, we present two novel cases of CS/EL with recessive mutations in ADAMTSL4 and confirm the genotype of a patient originally described by Cruysberg et al. [32] (Table 1).

The fact that multiple, generally unrelated organ systems are affected in FBN1-associated syndromes (MFS, WMS, etc.) has prompted much research on the mechanism of disease. Broadly, it has been suggested that disrupted FBN1 protein might have both primary (disintegration of fibrillin microfibrils) and secondary (aberrant TGFb pathway signaling) effects depending on the tissue [39, 40].

Fibrillin 1 makes up the majority of the ocular zonule proteome in the human eye [41], so it is no surprise that protein-altering FBN1 mutations cause disintegration of the zonule and ectopia lentis. Interestingly, mutations affecting cysteine residues are associated with a higher risk of EL [39, 42]. This can be explained by the fact that the cysteine residues in the calcium binding epidermal growth factor-like (cbEGF) domains of FBN1 form disulfide bonds with one another stabilizing the calcium binding sites, and in doing so, help protect the FBN1 protein against proteolysis [43]. However, complete FBN1 haploinsufficiency can also cause EL [40], suggesting that ocular zonules require full expression of proteolysis-resistant protein for optimal function. Given that not all FBN1-rich tissues are affected by FBN1 mutations in as severe a way indicates that zonules are somehow particularly susceptible to FBN1 disintegration. Ahram et al. suggest that this is because of the zonule's relatively slow turnover attributing to a “temporal onset weakness” [15].

Most other MFS phenotypes have been attributed to dysfunctional TGFb-pathway signaling. Beyond molecular, tissue-specific analyses of ligand/receptor activity, and downstream consequences [44–46], this mechanism is evident in the overlap between MFS and syndromes such as LDS and SGS. For example, LDS 1 and 2 are caused by mutations in TGFBR1 and 2, respectively, and also present with aortic aneurysm, disproportionately long limbs, arachnodactyly, pectus deformity, scoliosis, and joint laxity (among others) [47]. SGS is caused by mutations in SKI, a SMAD inhibitor and presents with arachnodactyly, pectus deformity, scoliosis, joint hypermobility, and aortic root dilation (among others) [48]. In fact, SGS was historically referred to as Marfanoid craniosynostosis due to its significant overlap in features with MFS in addition to the distinguishing CS phenotype. Incidentally, LDS and SGS do not typically manifest with any of the ocular phenotypes of MFS (except for blue sclera).

Of note, while CS is not considered a diagnostic feature of MFS, there are several cases of CS in individuals with MFS (especially eoMFS) and many more reports of craniofacial phenotypes (dolichocephaly, brachycephaly, etc) that might suggest underdiscovered rates of CS in MFS. Unfortunately, eoMFS has particularly high neonatal mortality rates associated with cardiovascular failure, and radiographic evaluations are rarely performed or reported.

**Figure 2:**
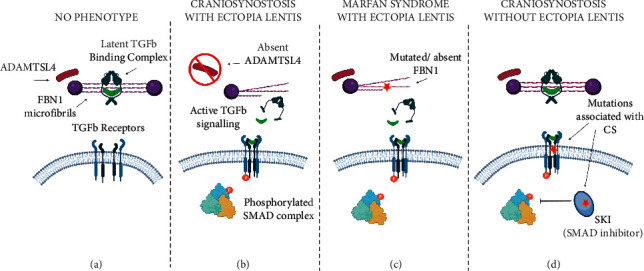
Schematic of proposed molecular mechanism of craniosynostosis and ectopia lentis phenotypes associated with ADAMTSL4, FBN1, and the TGFb pathway. (a) ADAMTSL4 is present and FBN1 is intact, TGFb ligand is tethered to fibrillin microfibrils, and the TGFb pathway is inactive. This does not cause a dysmorphic phenotype. (b) ADAMTSL4 is absent, fibrillin microfibrils are disintegrated leading to ectopia lentis, TGFb ligand is inappropriately released from extracellular matrix and binds TGFb receptors, and the TGFb pathway is aberrantly activated resulting in craniosynostosis. (c) ADAMTSL4 is present, but FBN1 is mutated, fibrillin microfibrils are disintegrated, TGFb ligand is inappropriately released from extracellular matrix and binds TGFb receptors, and the TGFb pathway is aberrantly activated resulting in Marfan syndrome with ectopia lentis. (d) ADAMTSL4 is present and fibrillin microfibrils are intact. Activating mutations in TGFb receptors and downstream modulators of the TGFb pathway cause aberrant signaling resulting in syndromic craniosynostosis (LDS/SGS) without ectopia lentis. (created with biorender.com).

We propose that, by the same processes as MFS-associated FBN1 mutations, recessive loss-of-function mutations in ADAMTSL4 cause ectopia lentis and craniosynostosis through primary (disintegration of fibrillin microfibrils) and secondary (aberrant TGFb pathway signaling) mechanisms, respectively (Figure 2). We know that ADAMTSL4 facilitates effective biogenesis and deposition of fibrillin microfibrils which make up the ocular zonules anchoring the lens to the ciliary body [31], and that almost all of the reported disease associated ADAMTSL4 variants lead to premature termination of the protein (HGMD) [49], suggesting that full-length, functional ADAMTSL4 protein is essential for creating and maintaining healthy ocular zonules.

Fibrillin microfibrils tether latent TGFb ligand through the latent TGFb binding protein complex thus regulating its release into the ECM (reviewed in [50]). It can further be hypothesized that loss of function of ADAMTSL4, leading to disintegration of fibrillin microfibrils, and subsequent aberrant release of TGFb ligand, could initiate a cascade of TGFb pathway signaling dysregulation. As it is well understood that the TGFb pathway is integral in calvarial bone and suture homeostasis [51–53], it follows that, in ADAMTSL4 expressing sutures, loss of function of ADAMTSL4 could contribute to a craniosynostosis phenotype.

We further propose that although ADAMTSL4 is widely expressed in the human body, molecules that may normally compensate for its loss are inactive or unavailable in the ocular zonule and the developing cranium, leading to such a tissue-specific phenotype. While FBN1 mutations traditionally affect a myriad of tissues including not only the craniofacial skeleton and the ocular zonules but the cardiovascular system, long bones, and skin, ADAMTSL4 mutations only seem to cause EL and EL with CS. There is no evidence of ADAMTSL4 mutations being associated with or acting as a modifier gene in other phenotypes. This suggests that in nonocular/calvarial systems that would normally be affected by FBN1 disintegration, there is likely a molecular reason that ADAMTSL4 loss does not cause a Marfanoid phenotype. Further investigation into tissue-specific mechanisms of microfibril biogenesis may uncover such an explanation.

It is notable that there is phenotypic variability associated with disease-causing ADAMTSL4 variants. For example, homozygosity of the 20 bp deletion leading to p.Gln256Profs*∗*38 is associated with both isolated EL/P and CS with EL/P in the same family (patient 2 and sibling) as well as isolated EL in other reports. However, there does not seem to be any evidence of a carrier phenotype in relatives with only one affected ADAMTSL4 allele.

In conclusion, we provide two more examples of ADAMTSL4-associated craniosynostosis with ectopia lentis to the literature and hypothesize that the molecular mechanism of this rare phenotypic overlap is likely highly correlated with that of FBN1-associated disease.

## Figures and Tables

**Table 1:** Patients with craniosynostosis and ectopia lentis reported to date.

Publication	Sex	Age CS	Age EL	Race/ethnicity	CS phenotype	EL phenotype	Gene affected	Nucleotide variant (s)	Protein variant (s)	Gnomad MAF
*Patients described in this paper*										
Patient 1	M	1 mo	4 mo	Caucasian	Bilateral coronal	Bilateral EL	ADAMTSL4	c.767_786del, c.2177 + 3_2177 + 6delGAGT	p.Gln256Profs*∗*38, (intronic - splice acceptor deletion)	0.001219, 0.000009137
Patient 2^+^	M	5 mo	Birth	Swedish	Sagittal	Bilateral EL/P	ADAMTSL4	c.767_786del, c.767_786del	p.Gln256Profs*∗*38, p.Gln256Profs*∗*38	0.001219

*Previously published ADAMTSL4-associated CS/EL patients*										
[28]	M	ND	ND	Norwegian	Sagittal^++^	EL/P	ADAMTSL4	c.767_786del, c.767_786del	p.Gln256Profs*∗*38, p.Gln256Profs*∗*38	0.001219
[27]	F	10 wk	10 mo	ND	Right coronal	Bilateral EL	ADAMTSL4	c.767_786del, c.767_786del	p.Gln256Profs*∗*38, p.Gln256Profs*∗*38	0.001219
[29, 32]	F	<2 yr	3 yr	Dutch	Metopic	Bilateral EL	ADAMTSL4	c.767_786del, c.2254C > T	p.Gln256Profs*∗*38, p.Gln752*∗*	0.001219, 0.000004059
[29, 32]	F	7 mo	3 yr	Dutch	Sagittal	Bilateral EL	ADAMTSL4	c.767_786del, c.2254C > T	p.Gln256Profs*∗*38, p.Gln752*∗*	0.001219, 0.000004059

*Previously published, genotypically unsolved (non-Marfanoid) patients with CS/EL*										
[36]	M	<6 yr	23 yr	ND	Coronal	Bilateral EL	Unknown			
[35]	F	Birth	4 mo	ND	Left coronal	Bilateral EL	Unknown			
[33]	ND	<6 yr	<6 yr	Turkish	Parietal/tempoparietal	Bilateral EL	Unknown			
[34]	M	<6 yr	<6 yr	French	Sagittal (scaphocephaly)	Bilateral EL	Unknown			

*Previously published patients with CS/EL as features of early-onset Marfan syndrome*										
[26]	F	<7 yr	<7 yr	ND	Scaphocephaly with craniosynostosis	EL	FBN1	c.3668G > A	p.C1223Y	Not listed
[25]	M	8 mo	6 yr	ND	Sagittal	Bilateral EL	FBN1	c.3302G > A	p.Y1101C	Not listed

M, male; F, female; ND, not described; CS, craniosynostosis; EL, ectopia lentis; EL/P, ectopia lentis et pupillae; MAF, minor allele frequency. Age indicates age at diagnosis of CS and EL, respectively. ^+^Sister of patient 2 has the same genotype but presents only with EL/P. ^++^Detail ascertained through personal correspondence with author.

## Data Availability

The data that support the findings of this study will be openly available upon publication of this manuscript through the ClinVar database under accession numbers SCV002025267 and SCV002025268.
